# Hypofractionated Radiotherapy as a Standalone Treatment Modality for Locally Advanced Type B2 Thymoma in an Octogenarian Patient: 45 Gy in 15 Fractions

**DOI:** 10.7759/cureus.51528

**Published:** 2024-01-02

**Authors:** Atsuto Katano, Yuki Kasuga, Shingo Ohira, Masanari Minamitani, Hideomi Yamashita

**Affiliations:** 1 Radiology, The University of Tokyo Hospital, Tokyo, JPN; 2 Comprehensive Radiation Oncology, The University of Tokyo, Tokyo, JPN

**Keywords:** hypofractionation, octogenarian, definitive treatment, radiotherapy, thymoma

## Abstract

Thymic epithelial tumors (TETs), particularly type B2 thymomas, are rare neoplasms primarily found in the anterior mediastinum. The current therapeutic approach includes surgery, chemotherapy, and radiotherapy, but there is limited research on radiotherapy as a standalone treatment. This case report aims to elucidate the clinical outcomes of hypofractionated radiotherapy as a standalone treatment for locally advanced type B2 thymoma, offering insights into its potential efficacy and role in clinical practice.

## Introduction

Thymic epithelial tumors (TETs) constitute a rare neoplastic entity primarily located in the anterior mediastinum, originating from epithelial cells within the thymus [1]. Within the spectrum of histological subtypes in TETs, type B2 thymomas stand out for their distinctive expression of cortical and medullary epithelial markers and are often more invasive than type A thymomas [2]. The current therapeutic approach typically involves a surgery-based strategy, incorporating chemotherapy and radiotherapy [3]. However, limited evidence supports non-surgical treatments, such as radiotherapy, alone.

This case report details a compelling instance of sustained remission achieved solely through hypofractionated radiotherapy in a patient diagnosed with a locally advanced type B2 thymoma. This report aims to provide valuable insights into the potential effectiveness of hypofractionated radiotherapy as a standalone treatment modality for locally advanced type B2 thymomas. The focus is on illuminating the clinical trajectory, treatment response, and long-term outcomes of this therapeutic approach. Through this contribution, we aim to enhance the understanding of the role of hypofractionated radiotherapy in the context of type B2 thymomas and its implications for clinical practice.

## Case presentation

An 82-year-old woman underwent a routine chest X-ray as part of a health checkup, revealing an abnormal shadow in the left pulmonary hilum. Subsequent chest computed tomography (CT) unveiled a tumor in the anterior mediastinum, infiltrating into the pulmonary arteries. The patient had a medical history of Parkinson's disease, diagnosed approximately 20 years prior, with a cerebral aneurysm and scoliosis. A CT-guided biopsy of the anterior mediastinal tumor was conducted, revealing a nodular proliferation of tumor cells with enlarged nuclei and pale eosinophilic cytoplasm encapsulated within fibrous tissue. Immunohistochemically, the infiltrating lymphocytes were positive for terminal deoxynucleotidyl transferase (TdT) while epithelial cells were negative for CD5. The findings indicated a World Health Organization (WHO) type B2 thymoma.

**Figure 1:**
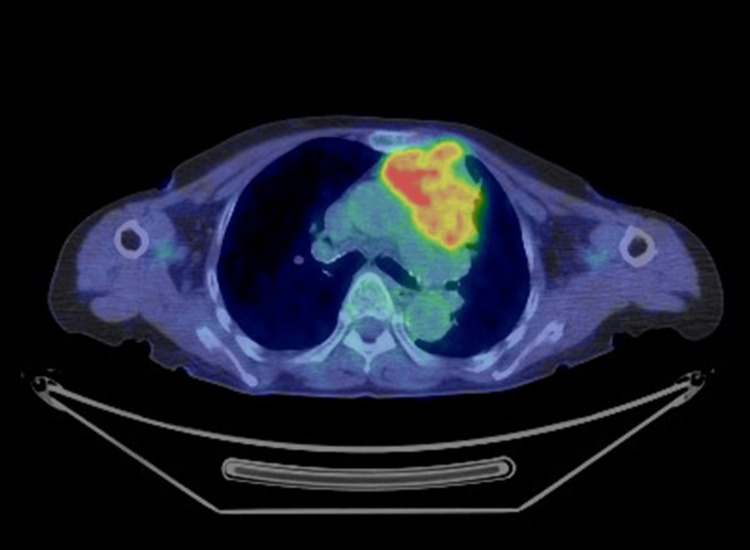
PET/CT scan A positron emission tomography/computed tomography (PET/CT) scan showed increased uptake in the anterior mediastinal lesion with a maximum standardized uptake value of 7.4.

A positron emission tomography-computed tomography (PET-CT) scan performed for staging showed increased uptake in the previously identified anterior mediastinal tumor measuring 80 mm in diameter with a maximum standardized uptake value (SUVmax) of 7.4 (Figure 1) and with no other abnormal uptake sites detected.

According to the 8th American Joint Committee on Cancer (AJCC) staging system, the clinical stage was T3N0M0 (Stage IIIA). Serologic test results revealed a slightly positive anti-acetylcholine receptor antibody (AChR-Ab) of 0.4 nmol/L.

Upon consultation with a multidisciplinary team, the patient was not considered a suitable candidate for surgery and chemotherapy due to her medical constraints and her expressed preference against surgery and chemotherapy. Hypofractionated radiation therapy was administered.

**Figure 2:**
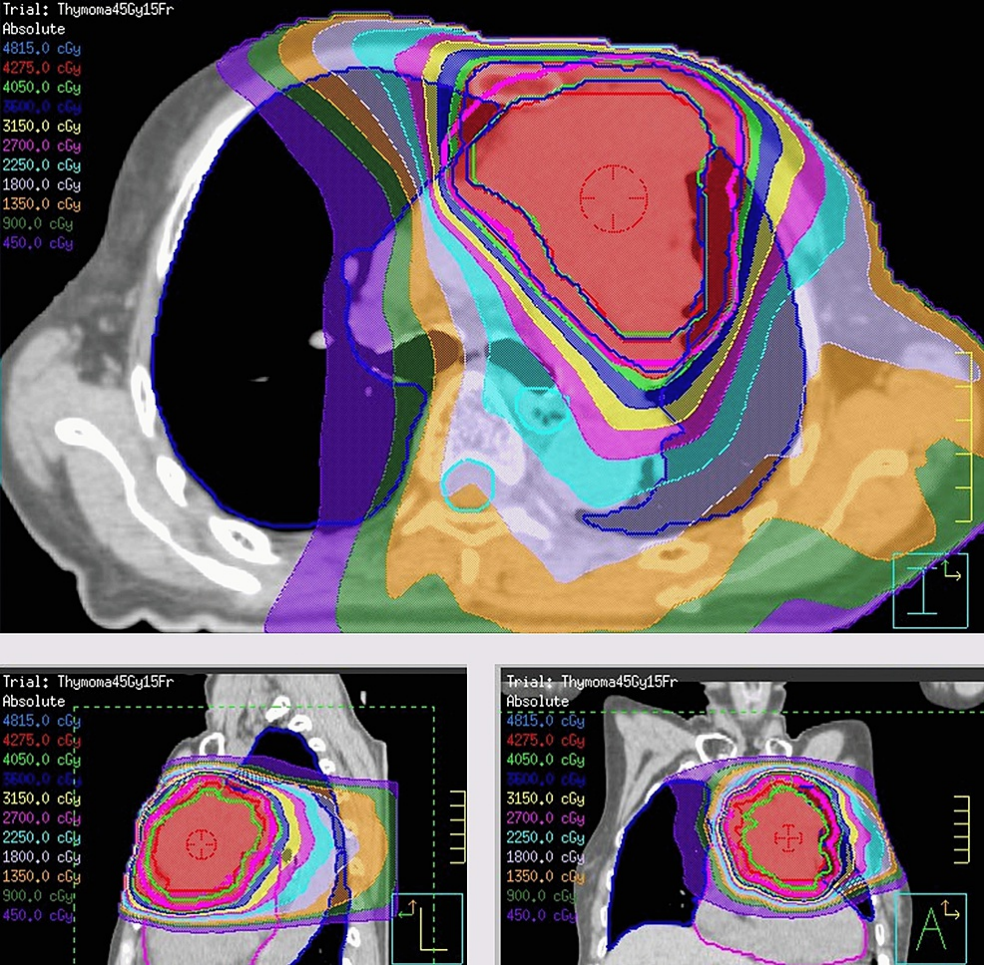
Dose distributions Dose distributions in the axial (upper), sagittal (lower left), and coronal (lower right) views for the radiotherapy plans

A planning CT for radiotherapy was acquired during the phases of inspiration and expiration. The gross tumor volume (GTV) was defined as the primary tumor, mapped out by the radiation oncologist using the PET-CT. The clinical target volume (CTV) was the GTV expanded by 5 mm, considering anatomical structure, and the internal target volume (ITV) was created by a combination of CTV in the inspiration and expiration phases. The planning target volume (PTV) was generated by additional expansion of the ITV plus 5 mm, considering mobility and daily setup errors. Elective nodal irradiation was not indicated. The patient was given 45 Gy in 15 fractions to cover 50% of PTV. The dose distribution is presented in Figure 2.

The dosimetric parameters are presented in Table 1.

**Table 1:** Dosimetric parameters for this radiotherapy PTV: planning target volume, D [X] %: minimum dose to X % of the target volume, V [X] Gy: the volume of normal organ receiving X Gy

Target	Parameter	Value
PTV	Volume	335.15 cc
	Volume of D98%	367.44 cc
	D98%	43.24 Gy
	D50%	45.00 Gy
	D2%	46.08 Gy
	Max	47.76 Gy
	Homogeneity index	0.06
	Conformity index	1.1
Lung	V20Gy	13.60%
	V10Gy	24.99%
	V5Gy	34.55%
	Mean	8.37 Gy
Heart	V40Gy	8.61%
	V30Gy	15.80%
	V20Gy	25.27%
	Mean	12.19 Gy
Spinal Cord	Max	19.72 Gy

**Figure 3:**
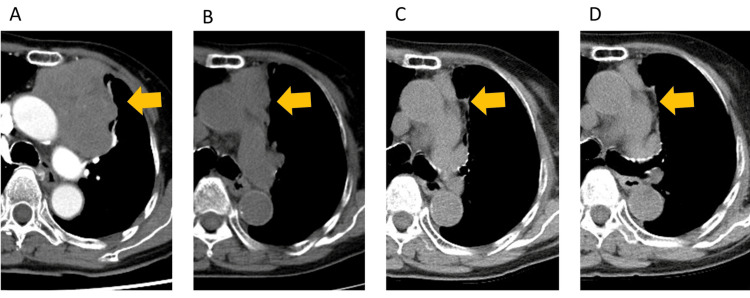
Chest CT scan Chest computed tomography (CT) scan before the radiotherapy (A), three months after (B), 16 months after radiotherapy (C), and 28 months after radiotherapy (D). Yellow arrows indicate the target lesion.

As an acute phase adverse event, grade 1 odynophagia and grade 1 pleuritis were experienced. After three months of radiotherapy, the lesion had shrunk on the CT scan, and the serum AChR-Ab level had normalized. As of 2.5 years later, it continues to show a sustained reduction in size with no apparent late-phase radiation-related adverse events (Figures 3A-3D).

## Discussion

Managing locally advanced type B2 thymoma presents a notable clinical challenge, especially in elderly patients burdened with comorbidities. This case introduces a distinctive strategy: utilizing hypofractionated radiotherapy as a standalone treatment modality in an octogenarian patient, resulting in sustained remission during an extended follow-up.

The treatment paradigm for TETs centers around surgery as the primary intervention, with radiotherapy typically reserved for postoperative therapy. In line with the European Society for Medical Oncology (ESMO) guidelines, chemotherapy followed by surgery or chemoradiotherapy is often recommended for unresectable thymic tumors [4]. The recently published Chinese guideline recommends chemoradiotherapy for unresectable TETs [5]. However, there has been minimal research on non-surgical treatment for TETs. A prospective phase 2 study aimed to assess the efficacy and safety of intensity-modulated radiation therapy (IMRT) combined with etoposide and cisplatin for patients with unresectable TETs [6]. The study enrolled 56 patients with limited advanced unresectable TETs; the objective response rate was 85.7%, with a median follow-up of 46 months. Their study suggests that IMRT with EP is effective and safe for limited advanced unresectable TETs, offering a viable treatment option for these patients. Fan et al. stressed the importance of radiotherapy for locally advanced, irresectable Masaoka-Koga stage III thymomas, evaluating the impact of definitive radiotherapy and debulking surgery [7]. Through multivariate analysis, they confirmed definitive radiotherapy as an independent beneficial prognostic factor for overall survival for irresectable Masaoka-Koga stage III thymoma patients.

In our present case, chemotherapy was contra-indicated, prompting the exclusive use of radiotherapy. This deviation from the conventional approach sheds light on the unexplored terrain of employing hypofractionated radiotherapy as a primary treatment for locally advanced thymomas, which remains relatively understudied in the existing literature.

Employing hypofractionation radiation schedules effectively minimized the risk of infection in radiation oncology during the coronavirus disease 2019 (COVID-19) pandemic [8]. Hypofractionated radiotherapy is now an accepted standard regimen for specific tumor sites such as prostate and breast cancer [9]. However, there is limited evidence for other malignancies. Our case contributes to this underexplored field by showcasing the feasibility and efficacy of hypofractionated radiotherapy. The discernible reduction in tumor size observed on follow-up imaging underscores the success of this approach. Notably, the patient's sustained remission over two years accentuates the potential of hypofractionated radiotherapy as an effective and well-tolerated treatment option. This is particularly significant for elderly patients with limited therapeutic alternatives.

## Conclusions

Hypofractionated radiotherapy proved to be effective in treating locally advanced type B2 thymoma in an elderly patient, demonstrating a notable absence of severe treatment toxicity. This limited toxicity has the potential to enhance the overall quality of life for these patients. The limitations of a single-case study suggest areas for future research, such as larger cohort studies or randomized trials, to validate these findings. This case contributes valuable insights into the potential efficacy and tolerability of hypofractionated radiotherapy, urging further research to establish its role as a treatment option for thymomas, particularly in elderly individuals with restricted therapeutic options.
